# Role of sinoatrial node architecture in maintaining a balanced source-sink relationship and synchronous cardiac pacemaking

**DOI:** 10.3389/fphys.2014.00446

**Published:** 2014-11-26

**Authors:** Sathya D. Unudurthi, Roseanne M. Wolf, Thomas J. Hund

**Affiliations:** ^1^Department of Biomedical Engineering, College of Engineering, The Ohio State UniversityColumbus, OH, USA; ^2^The Dorothy M. Davis Heart and Lung Research Institute, The Ohio State University Wexner Medical CenterColumbus, OH, USA; ^3^Department of Mathematics, The University of DubuqueDubuque, IA, USA; ^4^Division of Cardiovascular Medicine, Department of Internal Medicine, The Ohio State University Wexner Medical CenterColumbus, OH, USA

**Keywords:** sinoatrial node, automaticity, pacemaking, source-sink relationship, intercellular coupling, sick sinus syndrome, arrhythmia

## Abstract

Normal heart rhythm (sinus rhythm) depends on regular activity of the sinoatrial node (SAN), a heterogeneous collection of specialized myocytes in the right atrium. SAN cells, in general, possess a unique electrophysiological profile that promotes spontaneous electrical activity (automaticity). However, while automaticity is required for normal pacemaking, it is not necessarily sufficient. Less appreciated is the importance of the elaborate structure of the SAN complex for proper pacemaker function. Here, we review the important structural features of the SAN with a focus on how these elements help manage a precarious balance between electrical charge generated by the SAN (“source”) and the charge needed to excite the surrounding atrial tissue (“sink”). We also discuss how compromised “source-sink” balance due, for example to fibrosis, may promote SAN dysfunction, characterized by slow and/or asynchronous pacemaker activity and even failure, in the setting of cardiovascular disease (e.g., heart failure, atrial fibrillation). Finally, we discuss implications of the “source-sink” balance in the SAN complex for cell and gene therapies aimed at creating a biological pacemaker as replacement or bridge to conventional electronic pacemakers.

## Introduction

**Figure 1:**
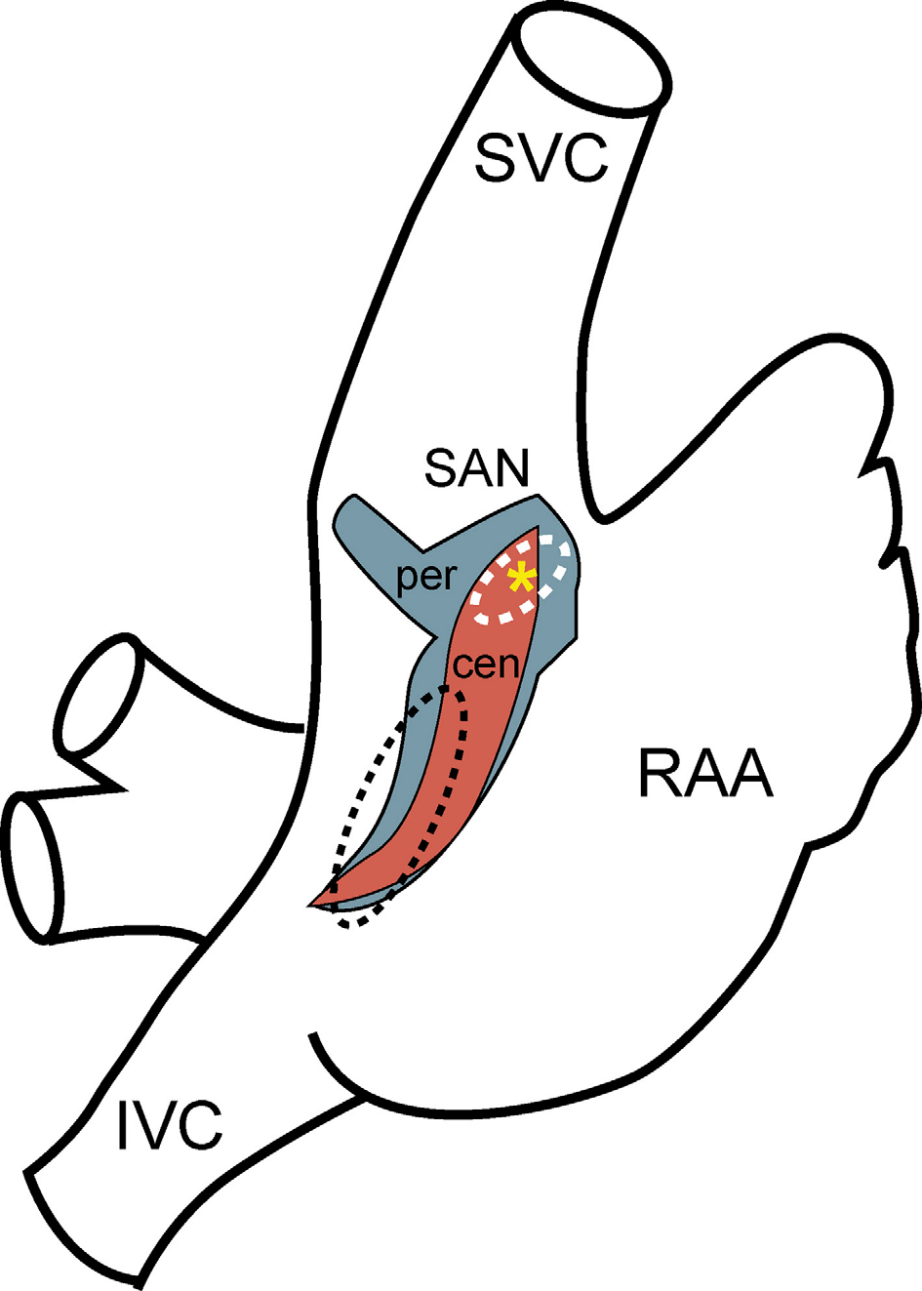
**Sinoatrial node anatomy**. Schematic diagram of the right atrium showing approximate location and extent of rabbit sinoatrial node determined by computer three-dimensional reconstruction (red, central SAN region; blue, peripheral SAN region; yellow asterisk, leading pacemaker site) compared to previous estimates from the human based on anatomy and function (white dashed line, SAN; black dashed line, ectopic atrial region) (Talano et al., 1978; Dobrzynski et al., 2005). (SVC, superior vena cava; IVC, inferior vena cava; RAA, right atrial appendage).

The sinoatrial node (SAN), located in the right atrium, serves as the primary site for initiation of the normal heartbeat (sinus rhythm) (Figure 1). Together with the cardiac conduction system [the atrioventricular node (AVN) and the His–Purkinje system], the SAN is responsible for orchestrating the precise sequence of electrical events underlying the normal heart rhythm. This sequence begins with generation of the electrical impulse in the SAN, which then spreads rapidly through the atria and to the AVN. AVN conduction is slow, thereby introducing a delay between atrial and ventricular systole to facilitate filling of the ventricles before they contract. The AVN also serves as a secondary pacemaker in the event of SAN failure (e.g., due to aging, cardiovascular disease). After exiting the AVN, the impulse propagates rapidly through the His-Purkinje system, comprised of a common bundle (the bundle of His), and left and right bundle branches that give rise to a network of terminal Purkinje fibers which simultaneously communicate the impulse to the ventricular muscle at multiple discrete locations (Purkinje-muscle junctions). The His–Purkinje system supports rapid conduction with action potential (AP) propagation velocities up to ten-fold higher than in the ventricular mass to ensure synchronous activation of the ventricular muscle (Boyett, 2009). This sequence of electrical events is essential for normal heart function and depends on a number of distinguishing cell and tissue properties shared by each component of the pacemaker and conduction system. First and foremost, cells in the pacemaker and conduction system possess the defining characteristic of automaticity, an ability to generate an action potential in the absence of an external stimulus. Automaticity is required for cardiac pacemaking and depends on a unique ion channel expression profile conducive to spontaneous AP generation. Second, and the major focus of this review, aside from active cell membrane properties (e.g., voltage-gated ion channel activity), tissue structure and cell-to-cell communication are tightly controlled in these tissues to handle the unique energetic demands placed on them during the excitation cycle. Specifically, these tissues are designed to balance the supply of depolarizing current generated by activating cells (“sources”) with the demand of downstream quiescent cells (“sinks”). Source-sink relationships are important throughout the heart, but tissues in the pacemaker and conduction system, in particular, have evolved elaborate structures/properties to facilitate activation of a large tissue mass by a relatively small number of cells (e.g., atrial activation by the sinoatrial node; ventricular activation by His-Purkinje fibers). The goal of this review is to discuss mechanisms important for regulation of cardiac pacemaking with a specific focus on the role of passive tissue properties.

## The sinoatrial node

### Sinoatrial node anatomy

The SAN was discovered over a century ago by Arthur Keith and Martin Flack as an anatomically defined tissue at the junction of the superior vena cava and right atria (Keith and Flack, 1907). For decades following this discovery, the SAN was defined as a compact unifocal region surrounded by secondary extranodal atrial pacemakers that could randomly compete with the leading pacemaker site in the SAN. Today, the SAN is recognized to be a distributed and heterogeneous complex adjacent to the crista terminalis with distinct regions defined by unique electrophysiological and structural properties (Figure 1) (Boineau et al., 1980, 1988; Dobrzynski et al., 2005, 2007; Fedorov et al., 2012). In larger animals, including the human, the node is functionally isolated with the exception of well-defined exit pathways that allow for communication between the SAN and atrial tissue (Fedorov et al., 2009, 2010, 2012; Nikolaidou et al., 2012). The anatomical basis of this insulation has been attributed to surrounding connective tissue, which insulates the pacemaker's automaticity from the hyperpolarizing electrical activity of the atrial myocardium. Other reports describe nodal/atrial interdigitations without a fibrous border that rely upon delicate spatial control of gap junction coupling (Ten Velde et al., 1995; Sanchez-Quintana et al., 2005). A distinguishing feature of SAN found in multiple species is the presence of a centrally located artery around which the SAN cells are organized (Sanchez-Quintana et al., 2005).

### Sinoatrial node cell morphology

**Figure 2:**
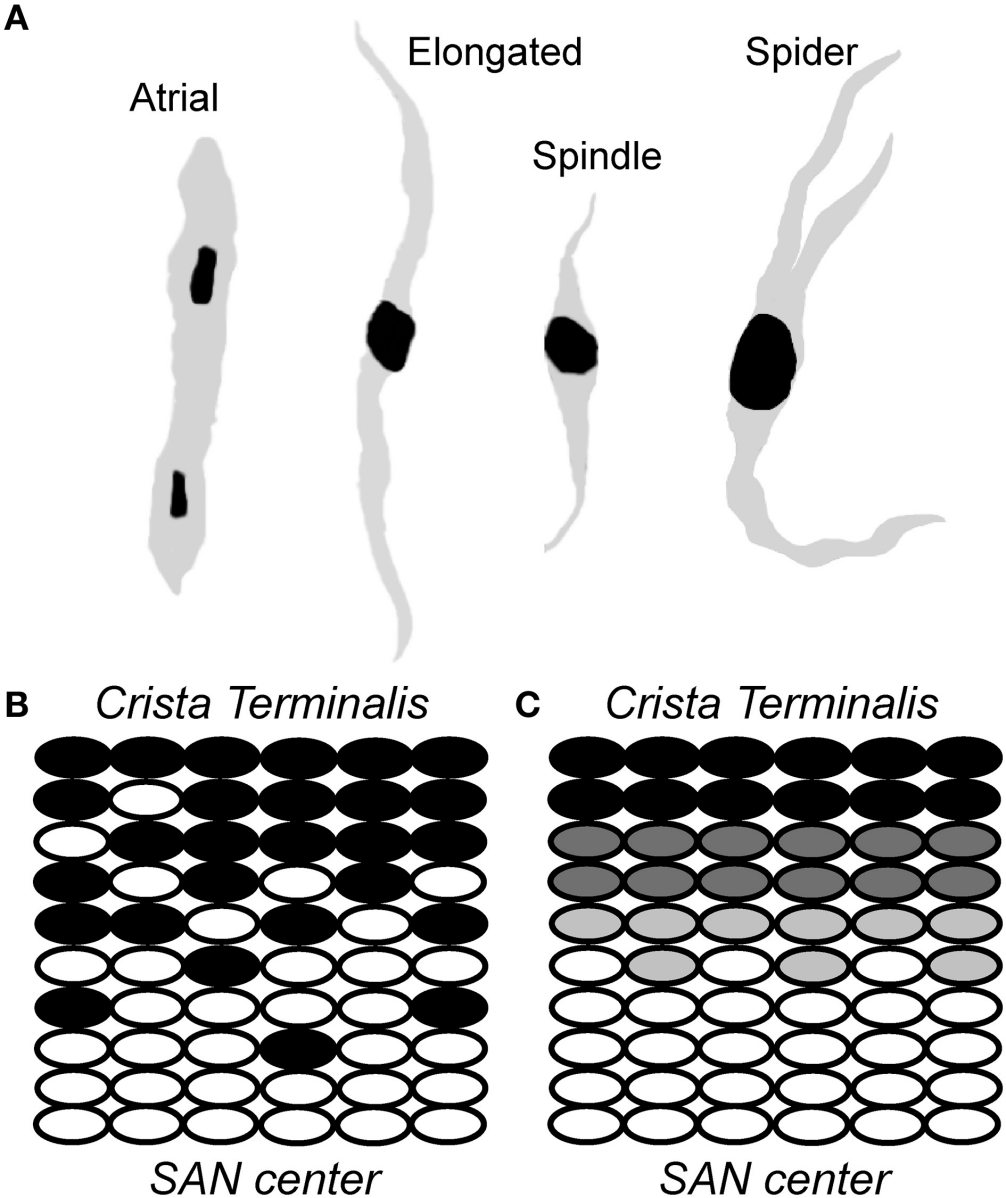
**Sinoatrial node is a heterogeneous collection of morphologically and electrophysiologically distinct myocytes**. **(A)** A schematic representation of cells found in the sinoatrial node region, including relatively large “elongated” spindle shaped cells, “spindle” cells that are similar in shape to that of elongated spindle cells with a shorter length, and irregularly shaped “spider” cells. **(B,C)** Two different models have been proposed to account for transition from typical/central SAN (white ovals) to atrial (black ovals) cells: **(C)** a gradual transition of intermediate cells (gray) from SAN center to crista terminalis; and **(B)** “mosaic” model involving gradually decreasing ratio of SAN to atrial cell densities from SAN center to crista terminalis (Verheijck et al., 1998).

The sinoatrial node is a collection of weakly coupled, heterogeneous cells, including pacemaker cells as well as non-pacemaker cells such as atrial myocytes, adipocytes and fibroblasts. Within the node, pacemaker cells vary by size and electrophysiological properties and may be divided into three major classes: (1) “Elongated spindle shaped cells,” which range up to 80 μm in length and have a faintly striated cell body with one or more nuclei; (2) “Spindle cells,” which have a similar shape to that of elongated spindle cells, but are shorter in length, extending up to 40 μm and are predominantly mono-nucleated; and (3) “Spider cells” have irregularly shaped branches with blunt ends (Verheijck et al., 1998) (Figure 2). A gradual transition has been observed in AP properties from the central area toward the SAN periphery (Kodama and Boyett, 1985). One of the possible reasons for this transition has been attributed to the arrangement and distribution of nodal cells in SAN node, although there is some debate about the exact underlying cellular distribution (Zhang et al., 2001; Dobrzynski et al., 2005; Oren and Clancy, 2010) (Figure 2). Regardless, it is clear that heterogeneity is the rule as none of the described cell types have been found exclusively in a specific SAN area. In rabbit, for example, a uniform distribution of the three major pacemaker cell types has been observed in the central SAN area. In the crista terminalis region, atrial cells are the predominant cell type (63 ± 18%), with a subpopulation of pacemaker cells, most of which are elongated spindle nodal cells. The septal area of SAN is mostly composed of atrial cells (88 ± 19%) and all the four types of SAN cells have an almost uniform presence in this region (Verheijck et al., 1998). Despite the diversity in morphology and electrophysiology within the SAN region, there are important unifying active membrane properties of these cells that are essential for normal pacemaking.

### Sinoatrial node cell active membrane properties

**Figure 3:**
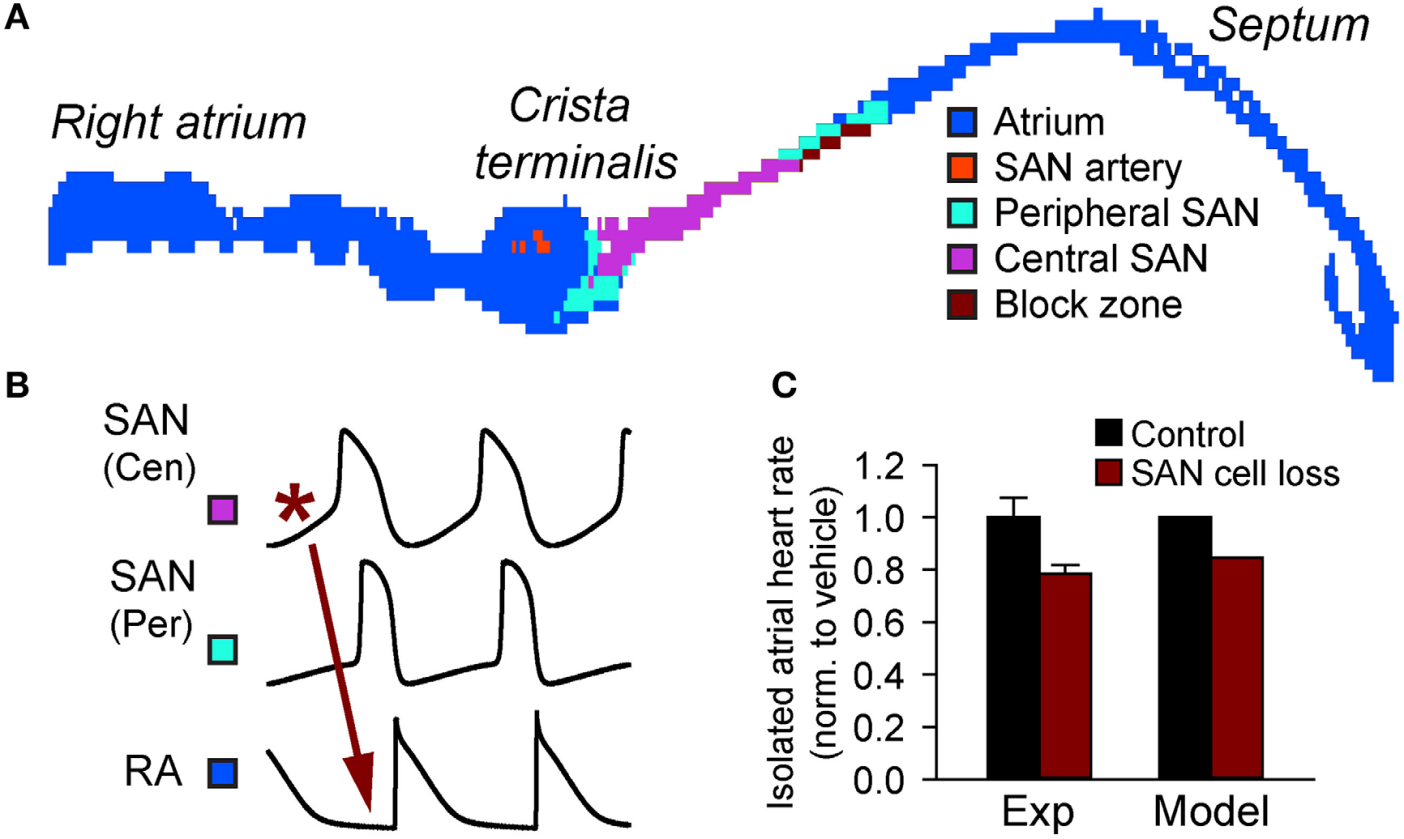
**Importance of source-sink balance in the intact sinoatrial node complex**. **(A)** A two-dimensional histologically reconstructed mathematical model of the intact sinoatrial node based on immunohistological data (Dobrzynski et al., 2005; Butters et al., 2010) identifying the distribution of cells throughout the right atrium (adapted from Luo et al., 2013). **(B)** Normal activation initiates in the central SAN region, spreads through the SAN periphery, and finally to the right atrium. **(C)** Cell loss slows activation of the right atrium in both experiment (19% cell loss induced by streptozotocin (STZ) treatment and assessed by TUNEL staining) and computer simulations (Replacement of 19% of SAN cells with inexcitable elements) (Luo et al., 2013).

SAN cell automaticity depends on an electrophysiological profile that is distinct from that in atrial or ventricular cells (Mangoni and Nargeot, 2008). First and foremost, while APs from ventricular and atrial cells possess a stable rest potential, the SAN AP lacks a stable rest potential due in large part to lack of the inward rectifier K^+^ channel *I*_*K*1_(Figure 3). Instead the SAN AP reaches a maximum diastolic potential (~-60 mV) followed by a spontaneous depolarization phase that eventually reaches threshold to generate another AP. Pacemaking depends on tight regulation of this spontaneous depolarization phase, which results from the coordinated effort of multiple ion channels/transporters/exchangers. Among the important currents contributing to diastolic depolarization is the hyperpolarization-activated “funny” current (*I*_*f*_) (due primarily to *HCN4* in SAN cells), with permeability to both Na^+^ and K^+^ and biophysical properties that make it a depolarizing current during diastole. *I*_*f*_ activates as the membrane potential approaches its max diastolic value and helps to spontaneously depolarize the membrane. At the same time, a low level of Ca^2+^ release from sarcoplasmic reticulum ryanodine receptor Ca^2+^ release channels likely promotes a depolarizing current via the Na^+^/Ca^2+^ exchanger (Lakatta et al., 2010). Once the membrane potential reaches about −50 mV, transient or T-type Ca^+2^ channels (namely, Ca_v_3.1, Ca_v_3.2, and Ca_v_3.3) open, allowing for Ca^2+^ entry into the cell and further depolarization of the cell membrane. As the membrane potential approaches −40 mV, L-type Ca^2+^ channels (first Ca_v_1.3, then Ca_v_1.2 at slightly less negative potentials) activate, giving rise to the SAN cell upstroke, which is much slower than that in atrial or ventricular myocytes due to the low levels of voltage-gated Na^+^ channel (Na_v_) expressed in SAN cells (orders or magnitude smaller than Na_v_ in ventricular myocytes). Although Na_v_ does not have the prominent role in the SAN AP upstroke, Na_v_ expression in SAN (and surrounding atrial) tissue is important for source-sink balance and pacemaking (discussed more below), such that Na_v_ deficiency is often associated with bradycardia and/or defects in the pacemaker and conduction system (Benson et al., 2003; Veldkamp et al., 2003; Makiyama et al., 2005). SAN AP repolarization is promoted by the activities of several classes of voltage-gated K^+^ channels. The transient outward K^+^ current (*I*_*to*_), carried by K_v_1.4, K_v_4.2, and K_v_4.3, is responsible for the early phase of SAN AP repolarization, while delayed-rectifier K^+^ currents ultrarapid (I_Kur_, K_v_1.5), rapid (I_Kr_, ERG), and slow (I_Ks_, K_v_LQT1) K^+^ currents control late repolarization and the maximal diastolic potential (especially *I*_*Kr*_). Other K^+^ currents (e.g., *I*_*K*,*ATP*_, *I*_*K*,*Ach*_) are important for dynamic response of SAN excitability to parasympathetic, metabolic factors. While steady-state rest is never achieved in the normal SAN, the activities of these K^+^ channels together with inactivation of L-type Ca^2+^ channels allows the membrane to reset for the next spontaneous action potential.

## Source-sink relationships in heart

While the ionic events described in the previous section are essential for SAN cell automaticity, cardiac pacemaking is, in fact, an emergent property of a heterogeneous, coupled system of both spontaneously active and quiescent cells (Figure 3). Therefore, a complete analysis of pacemaking accounts for not only events at the single cell level but also those tissue-level factors that will shape the emergent behavior. In particular, to understand the behavior of the intact SAN, it is helpful to reflect on the delicate electrical source-sink relationships that exist in the SAN region (Nikolaidou et al., 2012). Throughout the heart, successful propagation of the action potential depends on a cell first receiving electrical current from neighboring excited cells and then, once excited, sending current downstream to neighboring quiescent cells. Thus, every cell will both give charge to (“source”) and take charge from (“sink”) surrounding tissue at different times in their activation cycle. Propagation will be successful as long as the active sources can generate enough current to satisfy local sinks. Alternatively, if the sink overwhelms the source, propagation will fail. Tissue structure and electrical coupling have a huge influence on source-sink relationships, and can tip the balance for or against successful propagation at critical junctures (Joyner et al., 1991; Rohr et al., 1997; Shaw and Rudy, 1997). The concept of safety factor has been introduced to quantify the balance between sources and sinks in ventricular and atrial tissue (Delgado et al., 1990; Shaw and Rudy, 1997; Kleber and Rudy, 2004; Boyle and Vigmond, 2010). Briefly, safety factor is the ratio of how much charge a cell generates to the charge threshold for activation. A safety factor greater than one is indicative of safe conduction with an appropriate balance between source and sink, while a safety factor less than one is indicative of a source-sink mismatch with high susceptibility to conduction failure. Experimental and modeling studies have shown that safety factor tends to be low for propagation that occurs from a smaller cell to a larger cell and/or from a relatively small number of cells to a larger number of cells (Fast and Kleber, 1995; Rohr et al., 1997; Wang and Rudy, 2000), conditions that are highly relevant for the SAN complex. It is known that the atrium places a considerable electrical load on the SAN (Kirchhof et al., 1987). How, then, does the heart manage robust pacemaking under these unsafe conditions? The complete answer to this question cannot be understood without considering the SAN architecture itself.

## Sinoatrial node structure and source-sink balance

Perhaps the most important structural features for managing source-sink balance in SAN region are conduction barriers found between the SAN and surrounding atrial tissue (Fedorov et al., 2012; Nikolaidou et al., 2012). In large animals (e.g., canine and human), where the SAN is embedded in a relatively thick, three-dimensional atrial wall, layers of connective tissue isolate much of the SAN from the atrial myocardium (Fedorov et al., 2012; Nikolaidou et al., 2012). In smaller animals, the atrial wall is much thinner (practically two-dimensional) reducing the load on the SAN. However, even in this environment, a discrete region of conduction block (“block zone”) prevents direct activation of the interatrial septum and likely consists of fibrous, connective, and/or fatty tissue (Fedorov et al., 2012; Nikolaidou et al., 2012) (Figure 3). Thus, in both large and small animals well-defined non-conducting regions help reduce electrical loading of the SAN and preserve a sustainable source-sink balance.

Another important way source-sink balance is maintained in the SAN region is through electrical coupling between cells. Specifically, electrical coupling is poor in the SAN relative to that in the surrounding atrial myocardium due to preferential expression of gap junctions containing low conductance connexins (Connexin45) (Boyett et al., 2006; Dobrzynski et al., 2007). Modeling and experimental studies have demonstrated a somewhat counter-intuitive relationship between electrical coupling and safety factor, with uncoupling decreasing conduction velocity while increasing safety over a wide range (Shaw and Rudy, 1997; Rohr et al., 1998; Kleber and Saffitz, 2014). Partial uncoupling of SAN cells from neighboring cells prevents these cells from becoming overwhelmed by demand, in essence reducing the apparent size of the atrium (Joyner and Van Capelle, 1986; Watanabe et al., 1995). Importantly, experimental and modeling studies have shown that successful entrainment of atrial activation by the SAN requires only a low degree of coupling between SAN and atrial cells (Cai et al., 1994; Joyner et al., 1998). Once the electrical impulse emerges from the SAN, it can then propagate safely through the atria, into the AV node and eventually into the His-Purkinje system. Another region where a similar potential mismatch is observed is at the Purkinje muscle junction where the terminal Purkinje fibers couple to the ventricular mass. In fact, uncoupling has been shown to improve conduction across the Purkinje muscle junction (Morley et al., 2005). Aside from influencing safety for propagation, the degree of electrical coupling may also affect the dynamics of SAN cell spontaneous activation, with potential implications for synchronization of pacemaker activity (Glynn et al., 2014).

Finally, multiple experimental and modeling studies have demonstrated the importance of a gradient in membrane properties from the central to peripheral SAN region for normal pacemaking (Boyett et al., 2000; Zhang et al., 2007; Inada et al., 2014). Contributing to this gradient is the observed relationship between SAN cell size (or membrane capacitance) and densities of multiple ion currents, including voltage-gated Na^+^ and Ca^2+^ currents, as well as transient outward K^+^ current (*I*_*to*_), rapid and slow delayed rectifier K^+^ currents (*I*_*Kr*_, *I*_*Ks*_) and funny current (*I*_*f*_). Mathematical modeling has shown that, in particular, increased expression of the voltage-gated Na^+^ channel in the SAN periphery is necessary to support excitation of the atria by the SAN (Zhang et al., 2007).

Mounting experimental and modeling data support the notion that defects in SAN tissue architecture may tip the source-sink balance in a negative direction and promote sinus node dysfunction (Thery et al., 1977; Sanders et al., 2004; Dobrzynski et al., 2007; Fedorov et al., 2012; Nikolaidou et al., 2012). In fact, it has been known for almost 40 years that SAN structural changes (e.g., SAN cell loss and fibrosis) with aging is associated with sinus node dysfunction (Thery et al., 1977). Alterations in the normal gradient of electrophysiological properties in the SAN complex has been proposed as an additional mechanism for slowing of pacemaking with aging (Alings and Bouman, 1993). Besides the normal aging process, cardiovascular disease (e.g., heart failure) has also been shown to produce dramatic electrophysiological and structural remodeling in the atrium (Sanders et al., 2004; Dobrzynski et al., 2007). Increased expression of adenosine receptors within the SAN region together with increased fibrosis have recently been linked to loss of synchrony and sinus node dysfunction in a canine heart failure model (Lou et al., 2014). At the same time, experimental and modeling work has found that chronic angiotensin infusion (heart failure condition) in the mouse results in CaMKII-induced apoptosis resulting in SAN cell loss, which disrupts the normal source-sink balance leading to sinoatrial node dysfunction (Swaminathan et al., 2011) (Figure 3). A similar relationship between cell loss and sinus node dysfunction has been observed in a mouse model of diabetes and in ankyrin-B syndrome (Luo et al., 2013; Wolf et al., 2013).

## Implications of source-sink relationship for therapy

Studies from multiple groups have focused on development of a biological pacemaker as a replacement or bridge to traditional implantable electronic devices (reviewed in Cho and Marban, 2010; Rosen et al., 2011; Munshi and Olson, 2014; Rosen, 2014). These efforts are motivated by the limitations of electronic pacemakers, which include risk for infection (affecting ~2% of patients Rosen, 2014), lead fracture, and/or lack of autonomic responsiveness. Approaches include gene- and cell-based therapies to induce pacemaker activity from cells/regions that normally are quiescent (Miake et al., 2002; Bucchi et al., 2006; Tse et al., 2006; Zhang et al., 2011; Boink et al., 2012; Hu et al., 2014). Results have been mixed with reports of successful induction of physiologic heart rate using biological pacemaker in large animal models (Boink et al., 2013; Hu et al., 2014). Important limitations to these studies include both the magnitude and duration of the response to the intervention. For example, a recent gene therapy study used the transcription factor T-box 18 to reprogram right ventricular myocytes into pacemaker cells in a porcine heart block model *in vivo* (Hu et al., 2014). While authors reported a restored heart rate between 75 and 80 bpm in the T-box 18 transduced animals, there is some question about the exact mechanism as the adenoviral vector alone (GFP) also partially rescued heart rate (~65 bpm). Furthermore, heart rate effects in both the T-box 18 and GFP groups were transient and began to decline at time points greater than 11 days after injection (Hu et al., 2014; Rosen, 2014). While these biological approaches are compelling, the question is can we solve this challenging problem without considering the structure of the natural cardiac pacemaker? Is there a way to increase the efficacy of biological pacemakers by implanting/reprogramming them in supportive structures? Can we find a way to repair the natural pacemaker itself? New gene and cell-based therapies that address some of these questions may help fulfill the promise of an effective biological pacemaker.

## Conclusions

The SAN is an intricately designed structure tailored to support robust pacemaking, from the molecular composition of SAN cells to their spatial arrangement and connectivity to other cells. While cardiac pacemaking depends on the automaticity of the individual myocyte, architectural factors at the tissue level are also essential for robust pacemaking. This architecture involves a distinct SAN anatomy, a unique pattern of intercellular coupling, and gradients in electrophysiological profiles that help manage a very delicate source-sink balance, due to a very small number of cells having responsibility for activating a much larger number of cells. Importantly, structural remodeling of the SAN pacemaker complex is commonly associated with sinus node dysfunction (e.g., with aging, atrial fibrillation or heart failure). A major challenge going forward is to determine whether/how we can tune SAN architecture and the associated source-sink relationship for therapeutic benefit.

### Conflict of interest statement

The Associate Editor George E. Billman declares that, despite having collaborated with author Thomas J. Hund, the review process was handled objectively and no conflict of interest exists. The authors declare that the research was conducted in the absence of any commercial or financial relationships that could be construed as a potential conflict of interest.
